# Using deterministic record linkage to link ambulance and emergency department data: is it possible without patient identifiers? A case study from the UK

**DOI:** 10.23889/ijpds.v4i1.1104

**Published:** 2019-08-05

**Authors:** SJ Clark, M Halter, A Porter, HC Smith, M Brand, R Fothergill, SJ Lindridge, M McTigue, H Snooks

**Affiliations:** 1 Medical Directorate, London Ambulance Service, London. SE1 8SD; 2 Kingston University and St George’s, University of London, Centre for Health and Social Care Research, London SW17 0RE; 3 Swansea University Medical School, Singleton Park, Swansea SA2 8PP; 4 Department of Primary Care and Population Health, University College London, London, UK; Formally Nuffield Trust, 59 New Cavendish Street, London, UK; 5 Strategy Directorate, London Ambulance Service, London. SE1 8SD; 6 Clinical Audit and Research Unit, London Ambulance Service, London. SE1 0BW; 7 Clinical Trials Unit, Medical School, Warwick University Faculty of Health, Social Care and Education, Kingston University and St George’s, University of London, London SW17 0RE; 8 27 Devonshire Way, Croydon, CR0 8BU. Emergency Care Intensive Support Team, NHS Improvement, London, SE1 8UG; Formerly Medical Directorate, London Ambulance Service NHS Trust, London, SE1 8SD; 9 Operations West, London Ambulance Service, London. SE1 8SD

## Abstract

**Introduction:**

Routine linkage of emergency ambulance records with those from the emergency department is uncommon in the UK. Our study, known as the Pre-Hospital Emergency Department Data Linking Project (PHED Data), aimed to link records of all patients conveyed by a single emergency ambulance service to thirteen emergency departments in the UK from 2012-2016.

**Objectives:**

We aimed to examine the feasibility and resource requirements of collecting de-identified emergency department patient record data and, using a deterministic matching algorithm, linking it to ambulance service data.

**Methods:**

We used a learning log to record contacts and activities undertaken by the research team to achieve data linkage. We also conducted semi-structured interviews with information management/governance staff involved in the process.

**Results:**

We found that five steps were required for successful data linkage for each hospital trust. The total time taken to achieve linkage was a mean of 65 weeks. A total of 958,057 emergency department records were obtained and, of these, 81% were linked to a corresponding ambulance record. The match rate varied between hospital trusts (50%-94%). Staff expressed strong enthusiasm for data linkage. Barriers to successful linkage were mainly due to inconsistencies between and within acute trusts in the recording of two ambulance event identifiers (CAD and call sign). Further data cleaning was required on emergency department fields before full analysis could be conducted. Ensuring the data was not re-identifiable limited validation of the matching method.

**Conclusion:**

We conclude that deterministic record linkage based on the combination of two event identifiers (CAD and call sign) is possible. There is an appetite for data linkage in healthcare organisations but it is a slow process. Developments in standardising the recording of emergency department data are likely to improve the quality of the resultant linked dataset. This would further increase its value for providing evidence to support improvements in health care delivery.

**Highlights:**

- Ambulance records are rarely linked to other datasets; this study looks at the feasibility and resource requirement to use deterministic matching to link ambulance and emergency department data for patients conveyed by ambulance to the emergency department.
- It is possible to link these data, with an average match rate of 81% across 13 emergency departments and one large ambulance trust.
- All trusts approached provided match-able data and there was an appetite for data linkage; however, it was a long process taking an average of 65 weeks.
- We conclude that deterministic matching using no patient identifiers can be used in this setting.

## Introduction

The growing availability of electronically stored health data presents the opportunity to link otherwise isolated datasets [1, 2, 3]. Linkage of two or more separately-recorded pieces of information concerning a particular individual or family [4] has been used for health research purposes since the 1960s [5]. Increasingly, it is also used for auditing or to support improvements to patient care [6, 7, 8]. With enough common identifiers, health data can be linked to other data sources, such as social care, education and housing, with benefits felt across organisations and systems [9, 10, 11].

Successful implementation of data linkage across organisations is considered to demand high levels of community capital and inter-agency cooperation [11]. It has been estimated that 50-70% of the time and effort in real-world data mining projects is taken up with data preparation, compared with just 20-30% spent on data understanding [12]. In the UK, ambulance services and hospital trusts running emergency departments are separate organisations. Questions remain about the availability of linkable data within their routinely collected datasets and the appetite for, and capacity to, carry out data sharing.

Historically, in the UK, prehospital patient records from emergency ambulance services and hospital emergency department (ED) or inpatient records have not been routinely linked, though some recent research has begun to explore linkage [13, 14]. This is unsurprising, as the unique identifier for each patient (known as the National Health Service [NHS] number) is not commonly collected in ambulance records and thus there is no specific unique identifier available. Internationally, there are published examples of ambulance and ED data linkage achieved on a project basis, with a discrete extract of data [15, 16, 17]. There are also studies focussing on a particular condition, such as cardiac arrest [18], trauma [19], stroke [20] or myocardial infarction [21].

Pilot work in 2014 with one UK emergency ambulance service and one acute trust indicated that there was potential to use deterministic matching to link records [22]. Deterministic matching is a pass/fail system of data linkage in which corresponding records are either the same (a match) or not the same (not a match) [23]. Deterministic matching differs from probabilistic matching, which assigns degrees of agreement to corresponding records which are then considered a match if this agreement exceeds a threshold of similarity [23]. Probabilistic methods are generally favoured as they are not so sensitive to errors or slight differences in records that are otherwise a match [24]. Deterministic matching is most suited to datasets where an identifier is likely to have few or no errors, such as data generated automatically. This is considered a reasonably rare set of circumstances in data linkage [24], however, it did apply to the pilot dataset.

## Objectives

In this study we aimed to examine the feasibility of collecting de-identified emergency department patient record data and, using a deterministic matching algorithm, linking it to ambulance service data. Creating this dataset was part of a wider study known as PHED data (the Pre-Hospital Emergency Department Data Linking Project), which aimed to assess the potential benefits of retrospectively linking data from multiple hospital sites with ambulance service records in order to produce a dataset for analysis.

Five steps were previously identified in order to collect and link the data (senior approval, data availability; information governance; data transfer; data cleaning and linkage) [25]. Here, we report on these processes in detail, paying attention to linkage in a real-world situation where multiple healthcare providers are involved.

## Methods

### Design

We conducted a mixed-methods observational case study [26], gathering data from a learning log [27] kept by researchers and semi-structured interviews [28] with key stakeholders.

### Setting

The study was carried out in one ambulance trust and six acute NHS trusts in one UK metropolitan area. We documented all interactions required to create the linked data during the period 29/06/2015 to 07/11/2016. The linked data covered the period of 01/04/12 to 30/06/16, followed by on-going monthly extracts until the project closed on 15/09/17.

The ambulance trust recorded details of patient contact on paper records, which were subsequently scanned for electronic storage. The acute trusts managed a total of 13 EDs. To preserve anonymity, the six acute trusts are referred to as Trusts Alpha, Beta, Charlie, Delta, Echo and Foxtrot.

### Sample and participants

We aimed to approach a range of trusts based on publicly available information. They were selected in order to provide a range of: number of EDs [29]; number of overnight beds [30]; financial outturn 2013/14 [31]; Care Quality Commission rating [32]; ED four-hour access standard performance [33]; overall inpatient experience (out of 10) [34] and staff survey results [35].

For the interviews, we invited key information governance and information management staff involved in the linkage process from the ambulance trust and the acute trusts. Job roles included responsibility for business infrastructure, app or web development, and information governance.

**Figure 1: The flow of patients through the ambulance service to the Emergency Department (ED).**
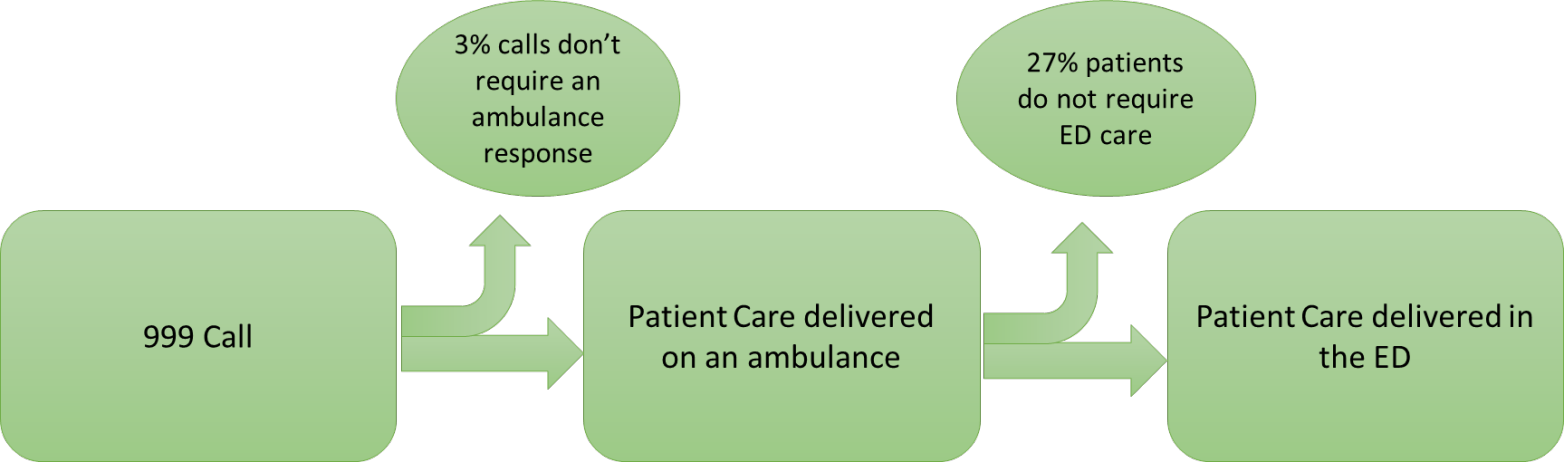


Figure 1 shows the flow of patients starting with the 999 call until receiving care in the ED. Patients whose care finished after their 999 call and those who did not require ED care (30% of patients in total; see Figure 1) were not included in the linked dataset.

### Patient and Public Representation

A patient and public panel, composed of 12 members recruited using an advertisement on the ambulance trust public-facing website and via the ambulance trust’s patient forum, met three times during the study. The patient and public panel offered ideas and advised on: the aims and objectives of the study; its conduct and progress; the research questions; project findings and outputs. Four further patient and public representatives were included in the study oversight group: two were members of the project board (which met quarterly) and two were members of the advisory group.

### Ethics and research governance approvals

The following approvals were granted: Health Research Authority approval to conduct the study (IRAS ID: 188336); research ethics approval from the Faculty of Health, Social Care and Education Research Ethics Committee of Kingston University and St. George’s, University of London; and research governance and information governance approvals from each of the seven participating NHS trusts.

### Data collection

Throughout the study, the research team maintained an electronic learning log. This recorded, as soon as possible, every task we carried out and interaction we had as part of the process leading to a linked dataset. The log was maintained in a Microsoft Excel 2013 spreadsheet with the following fields: nature of the occurrence or interaction; who was involved; duration; name of the acute trust; actions and any reflections. It did not include the acute trust staff time, as the learning log was completed by the research team only. The research team comprised a chief investigator, two researchers, an information governance manager and two information management staff.

We invited key staff involved in the process of data linkage (n=22) from the seven trusts to interview four months to one year after the data linkage was established. We sent email invitations, with up to three follow-up emails to those who did not respond. Semi-structured interviews were conducted at participants’ work premises. Our topic guide covered: gaining their perspective on the tasks involved in data extraction; transfer and linkage, issues related to information governance, and the potential value of linked data sets. Interviews were digitally audio recorded with participants’ consent and transcribed verbatim.

### The requested emergency department dataset

To generate a linked dataset, data from EDs were sought for the study time period. The variables requested from EDs were initially proposed by the research team on the basis of the pilot data linkage study [22] and agreed in discussion with acute trust staff. The data fields are presented in our supplementary material (Supplementary Appendix 1). Once a dataset was obtained from an ED, the process of linking those records to an ambulance record could take place. We requested a total of 18 data items from EDs, which were combined with 20data fields from the ambulance service. Since 7 data fields were recorded by both, this gave us a total of 31 different fields in our dataset.

### Data linkage

To undertake a deterministic matching approach a reliable identifier is required which is consistent across both datasets. In the absence of the unique identifier (NHS Number), the ambulance incident number (CAD) and the vehicle shift number (call sign) (Supplementary Appendix 1) generated at the point of the emergency call were used.

In order for these identifiers to be unique identifiers, dates and times were required. CAD number is automatically reset at midnight so the match requires a corroborating date, or consecutive dates if patient care crossed midnight. No other times were needed as CAD plus date alone is unique. Call sign is unique to each ambulance crew on a given shift, therefore the match requires a corroborating date (or consecutive dates if patient care crossed midnight) and corroborating arrival times at the ED, as the same crew could bring multiple patients to the same ED in a given shift. Times were never used as a standalone matching item.

Pilot work suggested that one or both of these event identifiers were collected in the emergency department along with a series of time stamps. CAD/call sign and date/time stamps are automatically generated by the ambulance trust, so it was assumed that the data entry error rate would be low. In the ED data, date/time stamps are also automatically generated. A full match deterministic matching algorithm was developed [23]. In order to be linked, an ED record must contain at least one event identifier. If both were available, n-1 deterministic linkage was applied, so that either event identifier could provide a match, but the CAD plus date algorithm was run first.

### Analysis

Learning log: we used summative content analysis [36] to cluster interactions and activities into a series of steps in the process of achieving a linked dataset. Descriptive statistics were calculated to show the number and type of interactions to complete each step, the time input of the project team and the duration in weeks for each step to be completed. The mean and median duration of activities across the acute trusts is provided.

Semi structured interviews: two researchers conducted a thematic analysis. Interview transcripts were read repeatedly, a coding framework was developed in discussion with the wider research team and applied to each transcript, and themes drawn out [37]. The coding framework for interviews can be found in Supplementary Appendix 2. We have used verbatim quotations to illustrate key points of agreement or dissent. NVivo (version 10) was used to manage the process of analysis.

## Results

### Participants

All six acute trusts approached agreed to participate in the study, alongside the ambulance service trust. Since some trusts manage multiple sites, 13 EDs were included. In total 958, 057 ED records were available for linking. The learning log contained 318 entries.

Of the 22 trust staff invited to interview, 12 had left or changed roles and five did not respond. Of the remaining five who agreed to take part in interviews, two worked for trust Charlie and three for the ambulance trust. Participants’ roles included responsibility for business infrastructure, app or web development, and information governance.

### The steps required to achieve and sustain a linked dataset

Tables 1 and 2 show the steps required to achieve and sustain a linked dataset, the total time required for each step per trust, as well as the overall, mean and median times required for each step. An initial preparatory phase of work within the ambulance service involved: gaining approvals; drafting a template information sharing agreement; agreeing on the data fields requested; developing a matching algorithm; and assigning a server with the appropriate security to receive the data transfer. These tasks were not related to any one acute trust and are recorded in the “ambulance” row of table 1.

We then identified five steps in the process required with each trust to achieve and sustain a linked dataset. These were: gaining senior approval for the study to take place; scoping data availability; negotiating information governance; data transfer; and data cleaning and linkage.

These steps were sequential, each depending on the completion of all previous steps within each trust. However, concurrent work occurred across different acute trusts.

Each step is now described in terms of its process, resource requirements and the perspectives of those involved. We indicate the total time taken by members of the research team in person hours for each step (Table 1).

**Table 1: Research team’s time (to the nearest hour) to complete each step of successful data linkage, by Trust:** 

Trust/Stage of data linkage process	1. Senior Approval	2. Data Availability	3. Information Governance	4. Data Transfer	5. Linking	Total
Ambulance	0	0	3	4	23	30
Alpha	5	3	8	3	2	21
Bravo	4	1	9	3	2	19
Charlie	5	4	6	8	23	46
Delta	4	1	10	4	8	27
Echo	26	4	4	1	8	43
Foxtrot	5	3	9	2	11	30

Grand Total	49	16	49	25	77	216
Mean across the acute trusts	8	3	8	4	9	23
Median across the acute trusts	5	3	9	3	8	28

### 1. Senior approval

Face to face meetings were held with representatives of each acute trust in order to secure senior approval. In five of the trusts, senior approval was granted during a single meeting, with some email communication before and after the meeting. One acute trust (Trust Echo) required separate approval at each of their sites, resulting in four meetings with four separate senior approvers. Senior approvers varied in their organisational designation and included medical directors, ED clinical leads, research leads, commissioners, operations managers, performance directors and directors of financial operations. Once approval was gained, the senior approver provided the contact details of the information manager and the information governance manager.

Two members of the research team attended each meeting, with an average of three members of acute trust staff. The total time involved for the research team in achieving senior approval was 48.5 person hours (2910 minutes). Gaining senior approval was one of the longer steps of the process, on average spanning 16.5 weeks to complete (see Table 2).

**Table 2: Time span (in calendar weeks) to complete each step per trust:** 

Trust/Stage of data linkage process	1. Senior Approval	2. Data Availability	3. Information Governance	4. Data Transfer	5. Linking	Total
Alpha	8	3	22	2	5	40
Bravo	3	4	14	10	6	37
Charlie	11	17	19	11	11	69
Delta	20	1	22	23	2	68
Echo	38	21	14	4	2	79
Foxtrot	19	43	25	9	1	97

Total	99	89	116	59	27	390
Mean	17	15	19	10	5	65
Median	15	11	21	10	4	69

### 2. Data availability

All six acute trusts were able to provide data for the majority of the 18 requested variables; however, data availability varied by trust, ranging from 14 variables (Trust Foxtrot) to 17 variables (Trusts Alpha and Charlie) (Table 3).

**Table 3: Availability of requested data from Emergency Departments (ED):** Notes: *CAD and call sign were in one free-text field without formatting. GP = General Practitioner; CAD = ambulance incident number.

ED variable requested	Availability of data by Trust					
	Alpha	Bravo	Charlie	Delta	Echo	Foxtrot
Hospital Site	Y	Y	Y	Y	Y	Y
ED Arrival date/time	Y	Y	Y	Y	Y	Y
Ethnicity	Y	Y	Y	Y	Y	Y
Age	Y	Y	Y	Y	Y	Y
Gender	Y	Y	Y	Y	Y	Y
GP Practice ID	Y	Y	Y	Y	Y	Y
FirstEDLocation	Y	Y	Y	Y	-	-
Blue Light Journey	Y	Y	Y	Y	Y	Y
Pathology	Y	-	-	-	Y	Y
Imaging	Y	-	Y	Y	Y	Y
Treatment	-	-	Y	Y	Y	Y
Diagnosis	Y	Y	Y	Y	Y	Y
Speciality Referral	Y	Y	Y	-	-	-
Outcome	Y	Y	Y	Y	Y	Y
Outcome Destination	Y	Y	Y	Y	Y	-
Date/Time of departure	Y	Y	Y	Y	Y	Y
Ambulance computer aided dispatch number	Y	*Y	Y	*Y	*Y	Y
Ambulance Call sign	Y	*Y	Y	*Y	*Y	-

In addition to variation in availability, there was variation in the recording and formatting of data. We developed a specification to ensure one matching algorithm could be used with all extracts on a routine basis. The specification included protocols for: file format; file names; column order; date/time conventions; column titles; absent data fields and list delineation. The specification required exact adherence, which meant some trusts required multiple revisions to the dataset they provided, ranging from one to nine revisions. Common errors included using “NULL” instead of “XXXX” for missing data fields, errors in the field names and re-ordering columns. Trust Charlie contained duplicates which were corrected by the acute trust’s information manager. However, more duplicates were found at data cleaning.

Work on data availability required three members of the research team. The time involved for the research team in negotiating data availability was 15.8 hours in total, with the range by trust (in hours) shown in table 1. Negotiating data availability was another long step of the process, on average spanning 14.8 weeks to complete (see table 2).

### 3. Information governance approval

All six acute trusts signed an information sharing agreement. Five acute trusts had very similar or identical terms within their information sharing agreements, three acute trusts used the project’s template and two acute trusts requested the use of their own template. Trust Foxtrot’s information sharing agreement was more extensive and included project specific clauses that were not included in a standard information sharing agreement. We worked with at least one information governance manager and one Caldicott Guardian (a senior member of staff in an NHS organisation that is ultimately responsible for protecting the confidentiality of patients’ health information) [38] from each NHS trust. The time involved for the research team to gain information governance approval was 48.5 hours in total, with the range by trust shown in table 1. Gaining information governance approval was the longest step of the process, on average taking 19.3 weeks to complete (see table 2). Time was taken to ensure that the terms of the agreements were comparable in order to allow the data from all sites to be subject to the same transfer, storage and analysis procedures.

### 4. Data transfer

**Figure 2: The process of data collection and linkage.**
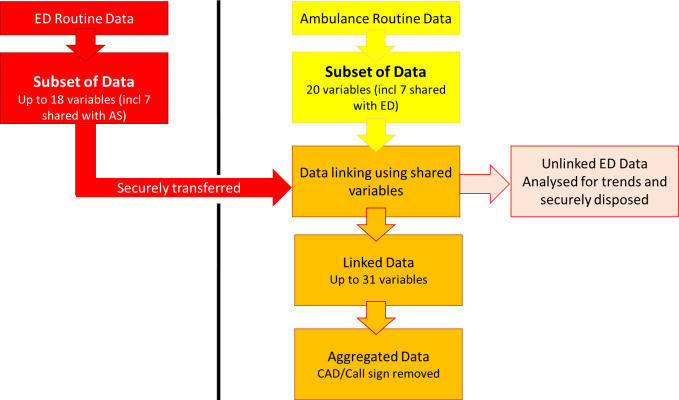
ED = Emergency Department; AS = Ambulance Service; CAD = ambulance incident number

All six acute trusts transferred data. The data were transferred either by Secure File Transfer Protocol onto a dedicated secure server or by encrypted email via nhs.mail accounts, whichever was most convenient for the trust (see figure 2). Test extracts were initially sent by an encrypted email account. Four acute trusts set up a monthly transfer via SFTP, and two acute trusts continued to send data via an encrypted email account.

Data transfer required two members of the research team plus at least one member of each acute trust’s information management team (generally the same people who scoped data availability). In addition, at least one member of each trust’s networks team and one from the ambulance service were required. The time involved for the research team in achieving data transfer was 24.6 hours in total, with the range by trust (in hours) shown in table 1. Achieving data transfer was a relatively short step of the process, on average taking 9.8 weeks to complete (see Table 2).

### 5. Data cleaning and linkage

The process of data collection and linkage is presented in Figure 2.

Data transfer from the six acute trusts formed an initial dataset of 958,057 records available for linkage, from 2012-2016 (disregarding ongoing transfer).

Data cleaning was required prior to linkage. This was particularly focussed on the event identifiers of CAD (a 1-4 digit number) and call sign (a 4 digit alpha numeric string), as these fields were required for linkage. All six acute trusts collected one or both event identifiers (see table 3). One acute trust (Trust Foxtrot) collected only CAD and three acute trusts collected CAD and call sign in one free-text box without formatting rules applied. For these three trusts, there was variation, both in the order and the completeness of CAD and call sign. However, there were patterns within the formats. Common formats included: ‘CAD123, Q987’; ‘123, Call sign Q987’; ‘Q987/123’. For these trusts, additional cleaning focussed on identifying 1-4 digit numerical strings (under 7000) for CAD and letters preceeding numbers for call sign. A list of all possible call signs was used to verify the call signs identified. Only exact matches were used.

Of the 958,057 records, 169,222 (17.7%) contained no information on either identifier and therefore could not be linked to an ambulance record. The remaining records were linked to an ambulance record giving an overall match rate of 81%, ranging from 50% to 94% across the trusts (see Table 4). CAD + date, which was run first, provided the most matches (n= 790,397 or 82.5%) with call sign providing an additional 167,660 records (17.5%).

**Table 4: Data completeness and linkage rate, by trust:** *(including 60 records of “136”)

Trust pseudonym	Number of records available for matching	Number of records with Nulls/Neither Identifier Available	Number of matched records	Match-rate
Alpha	>76,932	>38,782	>69,339	>90%
Bravo	>136,128	>19,854	>124,952	>92%
Charlie	>165,650	>148	>155,210	>94%
Delta	>193,528	>36,772	>138,984	>72%
Echo	>314,177	>23,922	>250,766	>80%
Foxtrot	>71,642	>49,744	>35,767	>50%
Total	>958,057	>169,222	>775018	>81%

Unmatched data were analysed for trends in time of day, age, CAD and call sign. One pattern was identified. In Trust Foxtrot’s CAD field, the number “136” was over-represented. Since the diagnosis field for records with a CAD 136 all contained a mental health diagnosis, we speculate that the CAD field was being used to report patients coming to the emergency department subject to section 136 of the Mental Health Act 1983, rather than the expected ambulance incident identifier.

Data linkage required two members of the research team and an information manager with specialist knowledge of the ambulance data system. The time involved for the research team in data linkage was 76 hours in total, with the range by trust shown in table 1. Data linkage took the research team’s most hours but was the shortest step of the process, requiring 4.5 weeks on average to complete.

### Perspectives of key staff involved in the process of data linkage

The key linkage staff shared perspectives on the process described above, which we present in three themes: feasibility of achieving data linkage, information staff motivation, and challenges to the processes.

### Feasibility

From the ambulance trust’s perspective, setting up the mechanisms for data linkage was seen as straightforward, in part because of the existing technical skills within the relevant team:

‘I think that the technical challenges are probably easier to solve because the guys doing it know what they’re doing. It’s not such an exotic thing to do, it’s just setting up an FTP server and the other end setting up something that can be run once a month or once a week to push the data through.’ WP1-03 Ambulance Service

The two representatives of Trust Charlie also saw the process of setting up data transfer as not at all onerous, taking roughly three work days over a period of two weeks, fitting around other work commitments. Once the process was set up, it required monthly transfers of data, taking about ten minutes each time. Trust Charlie was already engaged in setting up a data warehouse, so getting involved in PHED was ‘quite a straightforward project for us actually.’ WP1 -01b

Ambulance service stakeholders discussed the value of involving the right people in each acute trust, with day-to-day knowledge of the current system, especially if any trouble-shooting was required. In PHED data, the ambulance service took on the central facilitating role in the data linkage process, and ambulance service stakeholders described an iterative learning process, which became more efficient as the project progressed from trust to trust. The importance of acute trust buy-in at all managerial levels was emphasized to increase quality of the data at source.

Since the data linkage was carried out as part of an externally funded study, the ambulance service was able to allocate appropriate resources to support it:

‘there was funding for it, I just made sure that I dedicated my time to it, and yeah, we made it a priority, you know…funding helps.’ WP1-05 Ambulance Service

Other factors which enabled the linkage to be feasible included restricting access to the information, keeping identifiable data to an absolute minimum, the importance of having an experienced person in the ambulance service acting as liaison with the trusts and of seeing the benefit of data linkage, the latter being the next theme.

### Information staff motivation to achieve data linkage

The value of data linkage in general was emphasised by participants with prehospital to ED data linkage being just a small example of this practice. Benefits to patient groups, enhanced learning for clinicians, efficiency to systems and potential for money saving were reported. The value of data linkage included data linkage being “the right direction for the NHS as a whole” (WP1-05), the potential of data mining for uncovering correlations, making more informed decisions based on data and the synthesis of decision information systems.

Although there was enthusiasm for data linkage within the trusts in which our participants worked, they acknowledged that there could be different viewpoints within an acute trust, suggesting that it was important for senior decision makers to share a vision with their colleagues of the value of data linkage, if they were to support the time and effort involved in the process of linkage:

‘Some people were more enthusiastic about it than others, and that very much depended on their job title. And you may have a director of IT who doesn’t really – who just wants to have less work, you know, because it’s not a core business like running a hospital. Whereas you can have some researchers or some doctors or other clinicians who are involved in research who can see the benefit more.’ WP1-03 Acute trust

It was also suggested however that the senior decision maker may over-estimate the amount of work required, because they lack the specialist knowledge of the resource and expertise required, one of a number of challenges mentioned alongside the positive outlook.

### Challenges associated with the process of data linkage

While our participants had not described the actual process of linkage as onerous, they did still perceive time challenges including the capacity of staff with the specific skills required, ongoing maintenance requirements due to data failure or changing data, trouble-shooting and individual trust data cleaning.

From the ambulance service’s perspectives, there were also challenges to reaching agreements on information governance so that it satisfied the different perspectives of the various stakeholders across the six acute trusts:

‘We had to be quite careful about how we put the … information sharing agreement together, so everyone could agree with it. And we had quite a bit of to-ing and fro-ing between different trusts, getting it right so that it would suit each particular one in terms of where they were coming from.’ WP1-04 Ambulance Service

This resulted in ‘a lot of background preparation work’ (WP1-04) on top of the respondents’ main role.

After data transfer, discrepancies within and between datasets were recognised and needed to be resolved:

‘What we got back…did vary quite a lot from organisation to organisation… So for each organisation I had to build… an extra set of routines that would just work around trying to validate and, yeah, depending on what the quality of the data was.’ WP1-05 Ambulance Service

Ambulance service staff talked about the perceived advantages of standardising the process for data transfer across trusts, hoping it could be applied to any future data linkage work. This would minimise the labour involved in managing the discrepancies between different organisational systems and variations in data quality:

‘For this to succeed it would be good if there could be an agreed protocol [that] everyone could follow, and it’s generic enough so that every Trust’s IT department can follow it, just follow the instructions, basically’ WP1-03 Ambulance Service

Overall, the process was described as iterative, building on the experience from the pilot and learning from each trust, which enabled a more efficient process with the next trust. In summary the key enablers to support data linking for ambulance service and for EDs were: organisational buy-in and individual motivation; the right people with the right skills and capacity at the right time; liaison; and clear specifications. Standardised data were an anticipated benefit to the process.

## Discussion

### Summary of Key Findings

Our results show that collection and linkage of ED to ambulance data without NHS number is feasible across multiple sites, with a dataset of over three quarters of a million records and an overall match rate of 81%. We identified five necessary and sequential steps to this success. Negotiating senior approval translated into staff in information management and information governance roles completing the work required. All six trusts provided data that could be linked.

Processes were lengthy, on average taking over a year from requesting senior approval to achieving linkage, and not without challenges. Information governance took the most time and took the longest to complete, despite interview data reporting that pilot work had reduced initial set-up. The availability of the data fields was reasonably consistent across the sites, with omissions most likely for pathology/specialty referral. Interview data suggest that extraction and transfer from the acute trusts were reasonably simple steps, although trust-specific data cleaning prior to matching also added time and additional work. The technical aspects were not challenging, and the required skills were available in the ambulance service and acute trusts, but capacity is the issue.

Data linkage was possible with deterministic matching using CAD and call sign when they were clearly recorded. Inconsistencies in the ED data posed challenges to data linkage. Without mandatory recording of the ambulance identifiers in the ED, the match rate remains dependent on the consistency and accuracy of ED administrative staff. Blank records and the use of one free-text box for both CAD and call sign with no formatting rules applied at source were the main limiting factors to data linkage. This was not restricted to the fields required for linking: almost all the ED fields required additional data cleaning before meaningful data analysis was possible. Despite challenges, all trusts involved showed an appetite for this data linkage, with an eye for the potential benefits to patient care. Developing a common way of working across trusts appeared paramount to stakeholders as an enabler of simplified linkage and a useable resultant dataset.

### Study strengths and limitations

Our case study took a detailed approach to examining real-world processes of data linkage, with the learning log providing rigour for indicating the time and tasks entailed. We worked with six different acute trusts drawn from different areas within the ambulance catchment area. Our patient and public group actively engaged in discussions about the use of patient data in the study and issues of consent, and indicated their support for the approach taken by the study team.

This study had several limitations. We worked with only one ambulance trust, covering one of 11 ambulance trust regions in England, and with six acute trusts of the 236 acute trusts in England. This limits claims to generalisability. Where we achieved a lower than average match rate, we attributed this is to missing or mixed data in our identifier variables, and acknowledged this limitation to deterministic matching. We are also limited in the analysis of trends in the unmatched data. However, analysis of time of day, age, CAD and call sign found only one trend (involving 60 records with the CAD number 136) in the unmatched ED data. We acknowledge that further enquiry including statistical analysis may provide additional insights into improving the match rate. It would also improve the quality of the linked dataset as the variation in match rates between trusts has implications for research using the dataset. There may be current unknown selection biases caused by unknown absent patient groups found only in the unmatched records. Trusts with low match-rates (such as Trust Foxtrot) will be under represented in the dataset compared to those with high match-rates.

Not all patients cared for by the ambulance service will be taken to an ED, some will have their care resolved on the telephone or seen by the ambulance service and referred elsewhere, such as a GP or maternity unit. A match rate of 100% for all patients taken to the ED is unlikely to be achievable for practical reasons including: a minority of patients may walk out of the ED before they have been registered; multiple patients may be conveyed to the same ED in one ambulance; and human error such as a paramedic mistakenly inputting the wrong ED location. Trust Charlie, which had a match rate of 94% and only 148 records with null/neither identifier available, demonstrates that a high match rate is achievable if the ED data quality is high.

In addition, all data entered at ambulance call-taking are available electronically, however, the majority of the care delivered by pre-hospital clinicians and patients’ personal details remain in handwritten care records in this ambulance service, restricting the clinical data available for analysis. This may also limit the relevance of the findings to those ambulance trusts which have adopted electronic patient record systems. However, it is likely that the inclusion of data from electronic patient report forms would result in higher quality and more detailed data being available for analysis. Event identifiers were used to match the data, which had the added benefit of maintaining a pseudonymised dataset. However, the resulting data analysis cannot identify patients specifically. This restricts analyses looking at specific patients over time, potentially leading to certain patients being over-represented in the dataset while also making it impossible for this approach to be used to give feedback on specific patient outcomes to ambulance clinicians.

It is possible that using additional linking variables such as patient demographic and diagnostic similarities (alongside this study’s identifiers) may have increased the match rate. However Information and research governance requirements restricted testing of the matching method [24]. ED data could not be used to re-identify a patient in the ambulance routine data, which necessitated that staff with access to patient identifiable data in the ambulance record could not have access to CAD and call sign in the ED dataset. Without accessing identifiable data, we were not able to assess recall or precision or look at the prevalence of false non-links, a common weakness of deterministic matching. This meant that ED non-matches (the potential false non-links) could not be assessed against possible ambulance matches. While this may be seen as a limitation to those assessing data linkage purely, this is seen positively by those seeking to protect patients’ data used without explicit consent [39].

Data collection and transfer required a significant time investment over a prolonged period, from multiple members of the acute trust and the research team, across a wide range of expertise. We did not collect quantitative data on the number of people or time involved for the acute trusts. Although their qualitative feedback suggests this was protracted but not onerous, we are limited by the low response to our invitation to participate in interviews. Furthermore, the participants were limited to one acute trust (managing 2 EDs) and one ambulance trust, so the variations between the trusts could not be explored. While some of the low recruitment rate can be attributable to staff turnover, we cannot be sure that those who did not respond did not have a different experience to those interviewed.

### Interpretation in the context of other literature

Linkage of routine data across organisational boundaries is becoming increasingly common [8] and yet there are limited examples of ambulance data being linked routinely to ED data across all patient groups [11]. Our findings suggest that this linkage could be replicated by any UK ambulance service without a need to collect an additional unique identifier. Consequently, linking this data will provide greater potential for detailed analysis of clinical care beyond codes allocated by pre hospital clinicians.

Our finding that data linkage takes a long time is unsurprising in light of the challenges identified by Christen (2007) [12]. Christen (2007) identifies that the time investment required should not be underestimated, although the majority of this time-consuming work may have been a ‘one-off’ start-up cost with less input required on an ongoing basis [11]. Probabilistic matching is most commonly used in studies of this type as it takes account of potential inconsistencies in the variables used for linking [40]. One of the merits of this data linkage in particular is the high degree of certainty that a match exists. The patient pathway is linear, from ambulance to ED. Only patients that travelled to the ED were included in the ambulance data and only patients arriving by ambulance were included in the ED data.

Furthermore, in our study, the two primary linking variables, CAD and call sign, were relatively strong discriminating linkers and deterministic matching worked well. The result was 81% of ED records transferred were matched to a respective ambulance record. The prevalence of false non-links (real matches that are missed by the algorithm) is a common issue experienced in other research and can have a substantial impact on match rate. For example, studies linking this data internationally have match rates of 15% - 92% depending on the matching method and data available for matching [15, 16, 17].

CAD is especially powerful as it is unique to the date, and is a 1-4 digit number. It is quick and simple for both ambulance and ED staff to enter, compared to long string variables, such as name or address. Its brevity can however come with an increased risk of false links. Call sign is less reliable as a unique identifier, as multiple patients could arrive under the same call sign, albeit at different times. This is further exacerbated by hospital hand-over delays, making the time between patients difficult to estimate accurately.

While there is further work to be done to validate our method, we suggest that our match rate lends some support to arguments that deterministic linkage can be appropriate where the likelihood of the data match on a unique identifier is high.

### Implications

NHS England is in the early stages of implementing the Emergency Care Data Set [41], which started two months after the end of data collection for this project. This initiative provides a standardised list of data that each ED is required to collect, with the aim of reducing the differences in ED data collection and better understanding ED use. This dataset includes the mandatory and standardised collection of CAD and call sign, the absence of which was a significant limitation to linking in our study.

Standardised collection of CAD and call sign in the ED would also accommodate future provisions for ambulance data to be routinely linked. Implementing a small change in electronic data capture at the ED (two separate fields to capture CAD and call sign) plus the use of a deterministic matching method would yield high match rates. This would allow both local ambulance-to-ED linkage and the opportunity to include ambulance records in the national data repository [42], making it possible for ambulance data to be linked to multiple health datasets. Implications also exist for information governance, heeding the call from staff interviewed that standardised procedures would save resources. Nonetheless, this linked dataset was useable by the research team for analysis addressing topics including: ambulance telephone triage; GP use of the ambulance service; care of deteriorating patients; urgent care needs and ambulance commissioning. The data analysis was discussed with key stakeholders within the ambulance service and one acute trust.

## Conclusion

It is feasible to link ambulance and ED data without a unique patient identifier. The linkage process is currently lengthy and requires negotiation until pathways for linking are established and a single unique identifier is available in standardised data. Despite this, the linked dataset provides many opportunities for data analysis across a wide range of enquires, pertinent to research, clinical and commissioning arenas.

We present this as part of a wider project looking at the applications of the linked data. It is placed on a backdrop of international examples and other emerging pilot work in the UK. It is possible to share data and link it for research purposes, and this will only become easier with an increasing availability of standardised electronically collected health records.

## Supplementary Material

Please contact the corresponding author Sophie Clark for any supplementary material requests.
